# From event-labeled gene trees to species trees

**DOI:** 10.1186/1471-2105-13-S19-S6

**Published:** 2012-12-19

**Authors:** Maribel Hernandez-Rosales, Marc Hellmuth, Nicolas Wieseke, Katharina T Huber, Vincent Moulton, Peter F Stadler

**Affiliations:** 1 Max-Planck-Institute for Mathematics in the Sciences, Leipzig, D-04103, Germany; 2 Bioinformatics Group, Department of Computer Science; and Interdisciplinary Center of Bioinformatics, University of Leipzig, Leipzig, D-04107, Germany; 3 Center for Bioinformatics, Saarland University, Saarbrücken, D-66041, Germany; 4 High Throughput Bioinformatics, Faculty of Mathematics and Computer Science, Friedrich Schiller Universität Jena, Jena, D-07743, Germany; 5 Parallel Computing and Complex Systems Group, Department of Computer Science, University of Leipzig, Leipzig, D04103, Germany; 6 School of Computing Sciences, University of East Anglia, Norwich, NR4 7TJ, UK; 7 Inst. f. Theoretical Chemistry, University of Vienna, Vienna, A-1090, Austria; 8 Santa Fe Institute, Santa Fe, NM, 87501, USA

## Abstract

**Background:**

Tree reconciliation problems have long been studied in phylogenetics. A particular variant of the reconciliation problem for a gene tree *T *and a species tree *S *assumes that for each interior vertex *x *of *T *it is known whether *x *represents a speciation or a duplication. This problem appears in the context of analyzing orthology data.

**Results:**

We show that *S *is a species tree for *T *if and only if *S *displays all rooted triples of *T *that have three distinct species as their leaves and are rooted in a speciation vertex. A valid reconciliation map can then be found in polynomial time. Simulated data shows that the event-labeled gene trees convey a large amount of information on underlying species trees, even for a large percentage of losses.

**Conclusions:**

The knowledge of event labels in a gene tree strongly constrains the possible species tree and, for a given species tree, also the possible reconciliation maps. Nevertheless, many degrees of freedom remain in the space of feasible solutions. In order to disambiguate the alternative solutions additional external constraints as well as optimization criteria could be employed.

## Background

The reconstruction of the evolutionary history of a gene family is necessarily based on at least three interrelated types of information. The true phylogeny of the investigated species is required as a scaffold with which the associated gene tree must be reconcilable. Orthology or paralogy of genes found in different species determines whether an internal vertex in the gene tree corresponds to a duplication or a speciation event. Speciation events, in turn, are reflected in the species tree.

The reconciliation of gene and species trees is a widely studied problem [1-10]. In most practical applications, however, neither the gene tree nor the species tree can be determined unambiguously.

Although orthology information is often derived from the reconciliation of a gene tree with a species tree (cf. e.g. TreeFam [11], PhyOP [12], PHOG [13], EnsemblCompara GeneTrees [14], and MetaPhOrs [15]), recent benchmarks studies [16] have shown that orthology can also be inferred at similar levels of accuracy without the need to construct trees by means of clustering-based approaches such as OrthoMCL [17], the algorithms underlying the COG database [18,19], InParanoid [20], or ProteinOrtho [21]. In [22] we have therefore addressed the question: how much information about the gene tree, the species tree, and their reconciliation is already contained in the orthology relation between genes?

According to Fitch's definition [23], two genes are (co-)orthologous if their last common ancestor in the gene tree represents a speciation event. Otherwise, i.e., when their last common ancestor is a duplication event, they are paralogs. The orthology relation on a set of genes is therefore determined by the gene tree *T *and an "event labeling" that identifies each interior vertex of *T *as either a duplication or a speciation event. (We disregard here additional types of events such as horizontal transfer and refer to [22] for details on how such extensions might be incorporated into the mathematical framework.) One of the main results of [22], which relies on the theory of symbolic ultrametrics developed in [24], is the following: a relation on a set of genes is an orthology relation (i.e., it derives from some event-labeled gene tree) if and only if it is a cograph (for several equivalent characterizations of cographs see [25]). Note that the cograph does not contain the full information on the event-labeled gene tree. Instead the cograph is equivalent to the gene tree's homomorphic image obtained by collapsing adjacent events of the same type [22]. The orthology relation thus places strong and easily interpretable constraints on the gene tree.

This observation suggests that a viable approach to reconstructing histories of large gene families may start from an empirically determined orthology relation, which can be directly adjusted to conform to the requirement of being a cograph. The result is then equivalent to an (usually incompletely resolved) event-labeled gene tree, which might be refined or used as constraint in the inference of a fully resolved gene tree. In this contribution we are concerned with the next conceptual step: the derivation of a species tree from an event-labeled gene tree. As we shall see below, this problem is much simpler than the full tree reconciliation problem. Technically, we will approach this problem by reducing the reconciliation map from gene tree to species tree to rooted triples of genes residing in three distinct species. This is related to an approach that was developed in [26] for addressing the full tree reconciliation problem.

## Methods

### Definitions and notation

#### Phylogenetic trees

A *phylogenetic tree T (on L) *is a rooted tree *T *= (*V*, *E*), with leaf set *L *⊆ *V *, set of directed edges *E*, and set of interior vertices *V*^0 ^= *V*\*L *that does not contain any vertices with in- and outdegree one and whose root *ρ_T _*∈ *V *has indegree zero. In order to avoid uninteresting trivial cases, we assume that |*L*| ≥ 3. The ancestor relation ${≼}_{T}$ on *V *is the partial order defined, for all *x*, *y *∈ *V *, by $x{≼}_{T}y$ whenever *y *lies on the path from *x *to the root. If there is no danger of ambiguity, we will write x≼y rather than $x{≼}_{T}y$. Furthermore, we write x≺y to mean x≼y and *x *≠ *y*. For *x *∈ *V *, we write L(x):={y∈L|y≼x} for the set of leaves in the subtree *T *(*x*) of *T *rooted in *x*. Thus, *L*(*ρ_T _*) = *L *and *T *(*ρ_T _*) = *T *. For *x*, *y *∈ *V *such that *x *and *y *are joined by an edge *e *∈ *E *we write e=[y,x]ifx≺y. Two phylogenetic trees *T *= (*V*, *E*) and *T*' = (*V*', *E*') on *L *are said to be *equivalent *if there exists a bijection from *V *to *V*' that is the identity on *L*, maps *ρ_T _*to *ρ_T_*', and extends to a graph isomorphism between *T *and *T *'. A *refinement *of a phylogenetic tree *T *on *L *is a phylogenetic tree *T*' on *L *such that *T *can be obtained from *T*' by collapsing edges (see e.g. [27]). Suppose for the remainder of this section that *T *= (*V*, *E*) is a phylogenetic tree on *L *with root *ρ_T _*. For a non-empty subset of leaves *A *⊆ *L*, we define lca*_T _*(*A*), or the *most recent common ancestor of A*, to be the unique vertex in *T *that is the greatest lower bound of *A *under the partial order ${≼}_{T}$. In case *A *= **{***x*, *y***}**, we put lca*_T _*(*x*, *y*) := lca*_T _*(**{***x*, *y***}**) and if *A *= **{***x*, *y*, *z***}**, we put lca*_T _*(*x*, *y*, *z*) := lca*_T _*(**{***x*, *y*, *z***}**). For later reference, we have, for all *x *∈ *V *, that *x *= lca*_T _*(*L*(*x*)). Let *L' *⊆ *L *be a subset of |*L*'*| *≥ 2 leaves of *T*. We denote by *T *(*L*') = *T *(lca*_T _*(*L*')) the (rooted) subtree of *T *with root lca*_T _*(*L*'). Note that *T*(*L*') may have leaves that are not contained in *L*'. The *restriction *$T{|}_{L^{\prime }}$ of *T *to *L***^' ^**is the phylogenetic tree with leaf set *L*' obtained from *T *by first forming the minimal spanning tree in *T *with leaf set *L*' and then by suppressing all vertices of degree two with the exception of *ρ_T _*if *ρ_T _*is a vertex of that tree. A phylogenetic tree *T' *on some subset *L*' ⊆ *L *is said to be *displayed *by *T *(or equivalently that *T displays T*') if *T*' is equivalent with tree $T{|}_{L^{\prime }}$. A set T  of phylogenetic trees *T *each with leaf set *L_T _*is called *consistent *if T=∅ or there is a phylogenetic tree *T *on $L=\cup_{T\in T}L_{T}$ that *displays *T , that is, *T *displays every tree contained in T . Note that a consistent set of phylogenetic trees is sometimes also called compatible (see e.g. [27]).

It will be convenient for our discussion below to extend the ancestor relation ${≼}_{T}$ on *V *to the union of the edge and vertex sets of *T*. More precisely, for the directed edge *e *= [*u*, *v*] ∈ *E *we put $x{≺}_{T}e$ if and onfly if $x{≼}_{T}v$ and $e{≺}_{T}x$ if and only if $u{≼}_{E}x$. For edges *e *= [*u*, *v*] and *f *= [*a*, *b*] in T we put e≼f if and only if v≼b.

#### Rooted triples

Rooted triples are phylogenetic trees on three leaves with precisely two interior vertices. Sometimes also called rooted triplets [28] they constitute an important concept in the context of supertree reconstruction [27,29] and will also play a major role here. Suppose *L *= **{***x*, *y*, *z***}**. Then we denote by ((*x*, *y*), *z*) the triple *r *with leaf set *L *for which the path from *x *to *y *does not intersect the path from *z *to the root *ρ_r _*and thus, having. $\text{lc}{\text{a}}_{r}\left(x,y\right)≺\text{lc}{\text{a}}_{r}\left(x,y,z\right)$ For *T *a phylogenetic tree, we denote by ℜ(T) the set of all triples that are displayed by *T *.

Clearly, a set R  of triples is *consistent *if there is a phylogenetic tree *T *on $X=\cup_{r\in R}L\left(\rho_{r}\right)$ such that R⊆R(T). Not all sets of triples are consistent of course. Given a triple set R  there is a polynomial-time algorithm, referred to in [27] as BUILD, that either constructs a phylogenetic tree *T *that displays R  or that recognizes that R  is *inconsistent*, that is, not consistent [30]. Various practical implementations have been described starting with [30], improved variants are discussed in [31,32].

The problem of determining a maximum consistent subset $R^{\prime }$ of an inconsistent set of triples, on the other hand is NP-hard and also APX-hard, see [33,34] and the references therein. We refer to [35] for an overview on the available practical approaches and further theoretical results.

The BUILD algorithm, furthermore, does not necessarily generate for a given triple set R  a minimal phylogenetic tree *T *that displays R , i.e., *T *may resolve multifurcations in an arbitrary way that is not implied by any of the triples in R . However, the tree generated by BUILD is minor-minimal, i.e., if *T***' **is obtained from *T *by contracting an edge, *T***' **does not display R  anymore. The trees produced by BUILD do not necessarily have the minimum number of internal vertices. Thus, depending on R , not all trees consistent with R  can be obtained from BUILD. Semple [36] gives an algorithm that produces all minor-minimal trees consistent with R . It requires only polynomial time for each of the possibly exponentially many minor-minimal trees. The problem of constructing a tree consistent with R  and minimizing the number of interior vertices is NP-hard and hard to approximate [37].

### Event labeling, species labeling, and reconciliation map

A gene tree *T *arises through a series of events along a species tree *S*. We consider both *T *and *S *as phylogenetic trees with leaf sets *L *(the set of genes) and *B *(the set of species), respectively. We assume that **|***L***| ≥ **3 and **|***B***| ≥ **1. We consider only gene duplications and gene losses, which take place between speciation events, i.e., along the edges of *S*. Speciation events are modeled by transmitting the gene content of an ancestral lineage to each of its daughter lineages.

**Figure 1:**
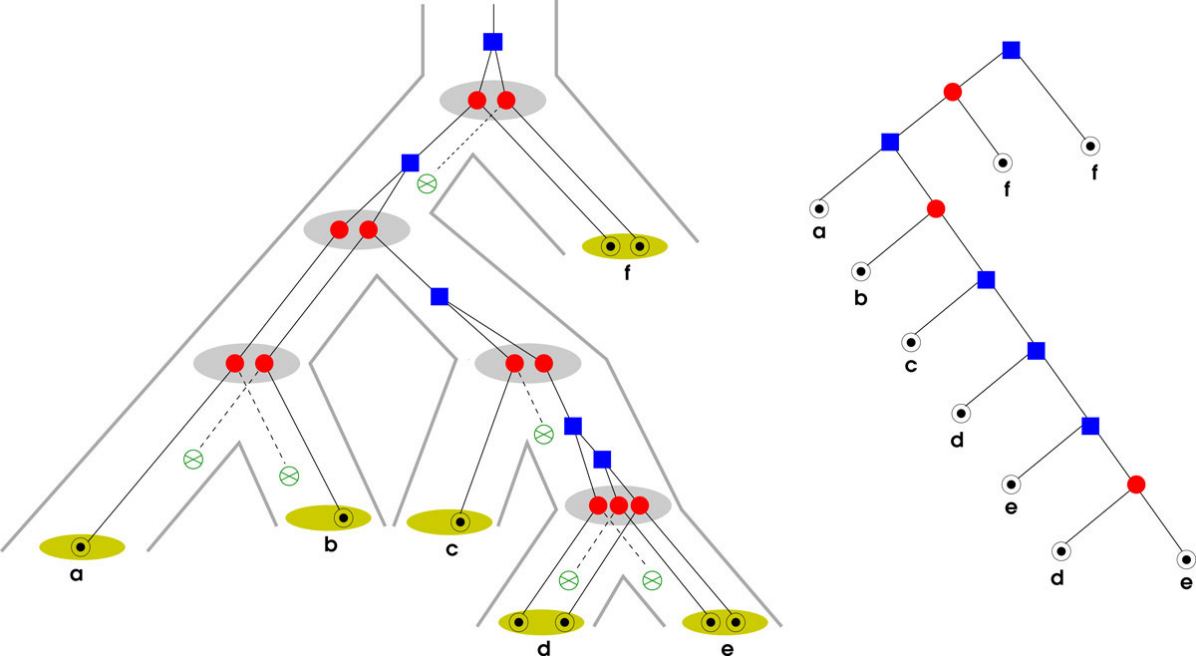
**Gene trees**. **Left: **Example of an evolutionary scenario showing the evolution of a gene family. The corresponding true gene tree $\hat{T}$ appears embedded in the true species tree Ŝ . The map $\hat{\mu }$ is implicitly given by drawing the species tree superimposed on the gene tree. In particular, the speciation vertices in the gene tree (red circuits) are mapped to the vertices of the species tree (gray ovals) and the duplication vertices (blue squares) to the edges of the species tree. Gene losses are represented with "⊗" (mapping to edges in Ŝ ). The observable species *a b*,*..*., *f *are the leaves of the species tree (green ovals) and extant genes therein are labeled with "⊙". **Right: **The corresponding gene tree *T *with observed events from the left tree. Leaves are labeled with the corresponding species.

The true evolutionary history of a single ancestral gene thus can be thought of as a scenario such as the one depicted in Figure 1. Since we do not consider horizontal gene transfer or lineage sorting in this contribution, an evolutionary scenario consists of four components: (1) A true gene tree $\hat{T}$, (2) a true species tree Ŝ , (3) an assignment of an event type (i.e., speciation •, duplication □, loss ⊗, or observable (extant) gene ⊙) to each interior vertex and leaf of $\hat{T}$, and (4) a map *µ *assigning every vertex of $\hat{T}$ to a vertex or edge of Ŝ  in such a way that (a) the ancestor order of $\hat{T}$ is preserved, (b) a vertex of $\hat{T}$ is mapped to an interior vertex of Ŝ  if and only if it is of type speciation, (c) extant genes of $\hat{T}$ are mapped to leaves of *S*. Alternatively, one could define $\hat{T}$ and Ŝ  to be metric graphs (i.e., comprising edges that are real intervals glued together at the vertices) with a distance function that measures evolutionary time. In this picture, $\hat{\mu }$ is a continuous map that preserves the temporal order and satisfied conditions (b) and (c).

In order to allow $\hat{\mu }$ to map duplication vertices to a time point before the last common ancestor of all species in Ŝ , we need to extend our definition of a species tree by adding an extra vertex and an extra edge "above" the last common ancestor of all species. Note that strictly speaking Ŝ  is not a phylogenetic tree anymore. In case there is no danger of confusion, we will from now on refer to a phylogenetic tree on *B *with this extra edge and vertex added as a species tree on *B *and to *ρ_B _*as the root of *B*. Also, we canonically extend our notions of a triple, displaying, etc. to this new type of species tree.

The true gene tree $\hat{T}$ represents all extant as well as all extinct genes, all duplication, and all speciation events. Not all of these events are observable from extant genes data, however. In particular, extinct genes cannot be observed. The observable part *T = T *(*V*, *E) *of $\hat{T}$ is the restriction of $\hat{T}$ to the leaf set *L *of extant genes, i.e., $T=\hat{T}{|}_{L}$.

Furthermore, we can observe a map σ: *L *→ *B *that assigns to each extant gene the species in which it resides. Of course, for *x *∈ *L *we have $\sigma \left(x\right)=\hat{\mu }\left(x\right)$. Here *B *is the leaf set of the extant species tree, i.e., *B *= σ(*L*). For ease of readability, we also put σ(*T*') = **{**σ(*x*): *x *∈ *L*(*y*)**} **for any subtree *T*' of *T *with *T*' = *T *(*y*) where *y *∈ *V°*. Alternatively, we will sometimes also write σ(*y*) instead of σ(*T *(*y*)). Last but not least, for *Y *⊆ *L*, we put σ(*Y *) = **{**σ(*y*): *y *∈ *Y***}**.

The observable part of the species tree *S = (W H) *is the restriction $Ŝ{|}_{B}$ of Ŝ  to *B*. In order to account for duplication events that occurred before the first speciation event, the additional vertex *ρ_S _*∈ *W *and the additional edge [*ρs*, lca*_s_B*] ∈ *H *must be part of *S*.

The evolutionary scenario also implies an *event labeling *map t:V→{∙,□,⊙} that assigns to each interior vertex *v *of *T *a value *t*(*v*) indicating whether *v *is a speciation event (**·**) or a duplication event (□). It is convenient to use the special label ⊙ for the leaves *x *of *T *. We write (*T*,*t*) for the event-labeled tree. We remark that *t *was introduced as "symbolic dating map" in [24]. It is called *discriminating *if, for all edges **{***u*, *v***} **∈ *E*, we have *t*(*u*) ≠ *t*(*v*) in which case (*T*,*t*) is known to be in 1-1-correspondence to a cograph [22]. Note that we will in general not require that *t *is discriminating in this contribution. For *T *= (*V*, *E*) a gene tree on *L*, *B *a set of species, and maps *t *and σ as specified above, we require however that *µ *and σ must satisfy the following compatibility property:

(C) Let *z *∈ *V *be a speciation vertex, i.e., *t*(*z*) = **·**, and let *T' *and *T" *be subtrees of *T *rooted in two distinct children of *z*. Then σ (*T*') **∩ **σ (*T"*) = ∅.

Note the we do not require the converse, i.e., from the disjointness of the species sets σ (*T*') and σ(*T"*) we do **not **conclude that their last common ancestor is a speciation vertex.

For *x*, *y *∈ *L *and *z *= lca*_T _*(*x*, *y*) it immediately follows from condition (C) that if *t*(lca*_T _*(*x*, *y*)) = **• **then σ(*x*) ≠ σ(*y*) since, by assumption, *x *and *y *are leaves in distinct subtrees below *z*. Equivalently, two distinct genes *x *≠ *y *in *L *for which σ(*x*) = σ(*y*) holds, that is, they are contained in the same species of *B*, must have originated from a duplication event, i.e., *t*(lca*_T _*(*x*, *y*)) = □. Thus we can regard σ as a proper vertex coloring of the cograph corresponding to (*T*, *t*).

Let us now consider the properties of the restriction of $\hat{\mu }$ to the observable parts *T *of $\hat{T}$ and *S *of Ŝ . Consider a speciation vertex *x *in $\hat{T}$. If *x *has two children *y*' and *y*" so that *L*(*y'*) and *L*(*y"*) are both non-empty then $x=\text{lc}{\text{a}}_{\hat{T}}\left(z^{\prime },\;z^{\prime \prime }\right)$ for all *z*' ∈ *L*(*y*') and *z" *∈ *L*(*y"*) and hence, *x *= lca*_T _*(*L*(*y*')∪(*L*(*y*")). In particular, *x *is an observable vertex in *T*. Furthermore, we know that $\sigma \left(L\left(y^{\prime }\right)\right)\cap \sigma \left(L\left(y^{\prime \prime }\right)\right)=\emptyset$, and therefore,$\hat{\mu }\left(x\right)=1\text{c}{\text{a}}_{S}\left(\sigma (L(y^{\prime }\right)\cup L\left(y^{\prime \prime }\right))$. Considering all pairs of children with this property this can be rephrased as $\hat{\mu }\left(x\right)=1\text{c}{\text{a}}_{Ŝ}\left(\sigma \left(L\left(x\right)\right)\right)$. On the other hand, if *x *does not have at least two children with this property, and hence the corresponding speciation vertex cannot be viewed as most recent common ancestor of the set of its descendants in *S*, then *x *is not a vertex in the restriction $T=\hat{T}{|}_{L}$ of $\hat{T}$ to the set *L *of the extant genes. The restriction *µ *of $\hat{\mu }$ to the observable tree *T *therefore satisfies the properties used below to define reconciliation maps.

**Definition 1**. *Suppose that B is a set of species, that S *= (*W*, *H*) *is a phylogenetic tree on B, that T *= (*V*, *E*) *is a gene tree with leaf set L and that σ *: *L ***→ ***B and *t:V→{∙,□,⊙}*are the maps described above. Then we say that S is a *species tree for (*T*,*t*, σ) *if there is a map µ *: *V ***→ ***W *∪ *H such that, for all x *∈ *V:*

*(i) If *t(x)=⊙*then µ *(*x*) = *σ *(*x*).

*(ii) If t*(*x*) = • *then µ *(*x*) ∈*W *\ *B*.

*(iii) If t*(*x*) = □ *then µ*(*x*) ∈ *H*.

*(iv) Let x*, *y *∈ *V with *$x{≺}_{T}y$. *We distinguish two cases:*

*1. If t*(*x*) = *t*(*y*) = □ *then *$\mu \left(x\right){≼}_{S}\mu \left(y\right)$*in S*.

*2. If t*(*x*) = *t*(*y*) = • *or t*(*x*) *≠ t*(*y*) *then *$\mu \left(x\right){≺}_{S}\mu \left(y\right)$*in S*.

*(v) If t*(*x*) = • *then µ*(*x*) = lca*_S_*(σ(*L*(*x*)))

*We call µ the reconciliation map from *(*T*,*t*, *σ *) *to S*.

We note that *µ*^-1^(*ρ_S_*) = ∅ holds as an immediate consequence of property *(v)*, which implies that no speciation node can be mapped above lca*_S_*(*B*), the unique child of *ρ_S_*.

**Figure 2:**
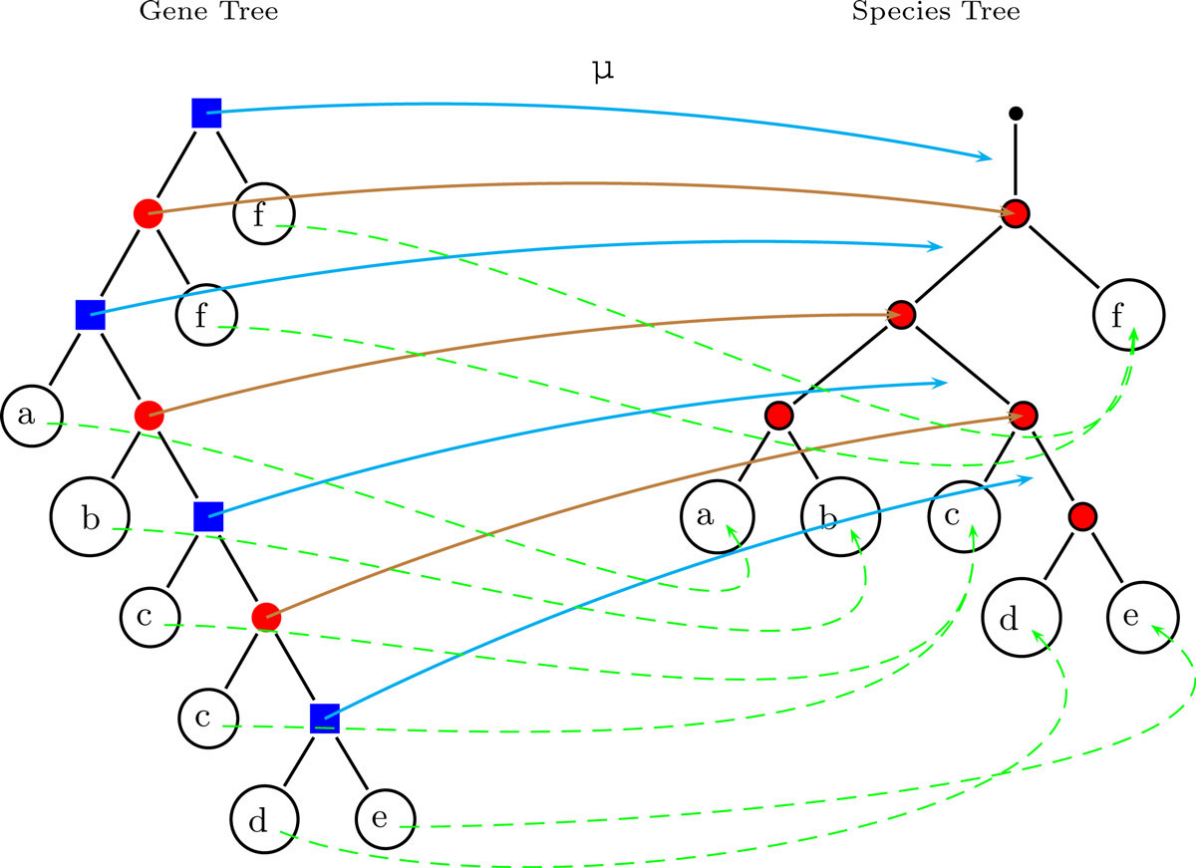
**Mapping *μ***. Example of the mapping *μ *of nodes of the gene tree *T *to the species tree *S*. Speciation nodes in the gene tree (red circles) are mapped to nodes in the species tree, duplication nodes (blue squares) are mapped to edges in the species tree. σ is shown as dashed green arrows. For clarity of exposition, we have identified the leaves of the gene tree on the left with the species they reside in via the map σ.

We illustrate this definition by means of an example in Figure 2 and remark that it is consistent with the definition of reconciliation maps for the case when the event labeling *t *on *T *is not known [38]. Continuing with our notation from Definition 1 for the remainder of this section, we easily derive their axiom set as

**Lemma 2**. *If µ is a reconciliation map from *(*T*,*t*, σ) *to S and L is the leaf set of T then, for all x *∈ *V*.

*(D1) x *∈ *L implies µ *(*x*) = *σ *(*x*).

*(D2.a) µ*(*x*) ∈ *W implies µ *(*x*) = lca*_S_*(σ (*L*(*x*))).

*(D2.b) µ *(*x*) ∈ *H implies *$\text{lc}{\text{a}}_{S}\left(\sigma \left(L\left(x\right)\right)\right){≺}_{S}\mu \left(x\right)$.

*(D3) Suppose x*, *y *∈ *V such that *$x{≺}_{T}y$. *If µ *(*x*), *µ *(*y*) ∈ *H then *$\mu \left(x\right){≼}_{S}\mu \left(y\right)$; otherwise $\mu \left(x\right){≺}_{S}\mu \left(y\right)$.

*Proof*. Suppose *x *∈ *V*. Then (D1) is equivalent to *(i) *and the fact that t(x)=⊙ if and only if *x *∈ *L*. Conditions *(ii) *and *(v) *together imply (D2.a). If *µ *(*x*) ∈ *H *then *x *is duplication vertex of *T*. From condition *(iv) *we conclude that $\text{lc}{\text{a}}_{S}\left(\sigma \left(L\left(x\right)\right)\right){≼}_{S}\mu \left(x\right)$. Since lca*_S_*(σ(*L*(*x*))) ∈ *W*, equality cannot hold and so (D2.b) follows. (D3) is an immediate consequence of *(iv)*. □

For *T *a gene tree, *B *a set of species and maps σ and *t *as above, our goal is now to characterize (1) those (*T*,*t*, σ) for which a species tree on *B *exists and (2) species trees on *B *that are species trees for (*T*,*t*, σ).

## Results and discussion

### Results

Unless stated otherwise, we continue with our assumptions on *B*, (*T*,*t*, σ), and *S *as stated in Definition 1. We start with the simple observation that a reconciliation map from (*T*,*t*, σ) to *S *preserves the ancestor order of *T *and hence *T *imposes a strong constraint on the relationship of most recent common ancestors in *S*:

**Lemma 3**. *Let µ *: *V *→ *W *∪ *H be a reconciliation map from *(*T*,*t*, σ) *to S. Then*

(1) $$1\text{c}{\text{a}}_{S}\left(\mu \left(x\right),\mu \left(y\right)\right){≼}_{S}\mu \left(1\text{c}{\text{a}}_{T}\left(x,y\right)\right)$$

*holds for all x*, *y *∈ *V*.

*Proof*. Assume that *x *and *y *are distinct vertices of *T*. Consider the unique path *P *connecting *x *with *y*. *P *is uniquely subdivided into a path *P*' from *x *to lca*_T _*(*x*, *y*) and a path *P*" from lca*_T _*(*x*, *y*) to *y*. Condition (iv) implies that the images of the vertices of *P*' and *P*" under *µ*, resp., are ordered in *S *with regards to ${≼}_{S}$ and hence are contained in the intervals $Q^{\prime }$' and $Q^{\prime \prime }$ that connect *µ*(lca*_T _*(*x*, *y*)) with *µ*(*x*) and *µ*(*y*), respectively. In particular *µ*(lca*_T _*(*x*, *y*)) is the largest element $\left(\text{w}\text{.r}\text{.t}.{≼}_{S}\right)$ in the union of $Q^{\prime }\cup Q^{\prime \prime }$ which contains the unique path from *µ*(*x*) to *µ*(*y*) and hence also lca*_S_*(*µ*(*x*), *µ*(*y*)).   □

Equation (1) is well known to hold for gene tree/species reconciliation in the absence of a prescribed event labeling in *T*.

Since a phylogenetic tree (in the original sense) *T *is uniquely determined by its induced triple set ℜ(T), it is reasonable to expect that all the information on the species tree(s) for (*T*,*t*, σ) is contained in the images of the triples in ℜ(T) (or more precisely their leaves) under σ. However, this is not the case in general as the situation is complicated by the fact that not all triples in ℜ(T) are informative about a species tree that displays *T*. The reason is that duplications may generate distinct paralogs long before the divergence of the species in which they eventually appear. To address this problem, we associate to (*T*,*t*, σ) the set of triples

(2) $$G=G\left(T,\;t,\;\sigma \right)=\left\{r\in ℜ\left(T\right)|t\left(\text{lc}{\text{a}}_{T}\left(r\right)\right)=∙\;\text{and}\;\sigma \left(x\right)\neq \sigma \left(y\right),\;\text{for}\;\text{all}\;x,y\in L\left(r\right)\;\text{pairwise}\;\text{distinct}\right\}.$$

As we shall see below, G(T,t,σ) contains all the information on a species tree for (*T*,*t*, σ) that can be gleaned from (*T*,*t*, σ).

**Lemma 4**. *If µ is a reconciliation map from *(*T*,*t*, σ) *to S and *((x,y),z)∈G(T,t,σ)*then S displays *((σ(*x*), σ(*y*)), σ(*z*)).

*Proof*. Put G=G(T,t,σ) and recall that *L *denotes the leaf set of *T*. Let $\left\{x,y,z\}\in ({}_{3}^{L}\right)$ and assume w.l.o.g. that ((x,y),z)∈G. First consider the case that *t*(lca*_T _*(*x*, *y*)) = •. From condition *(v) *we conclude that *µ*(lca*_T _*(*x*, *y*)) = lca*_S_*(σ(*x*), σ(*y*)) and *µ*(lca*_T _*(*x*, *y*, *z*)) = lca*_S_*(σ(*x*), σ(*y*), σ(*z*)). Since, by assumption, $\text{lc}{\text{a}}_{T}\left(x,y\right)≺\text{lc}{\text{a}}_{T}\left(x,y,\;z\right)$, we have as a consequence of condition *(iv) *that $\mu \;(\text{lc}{\text{a}}_{T}\left(x,y\right))≺\mu \left(\text{lc}{\text{a}}_{T}\left(x,y,\;z\right)\right)$. From lca*_T _*(*x*, *z*) = lca*_T _*(*y*, *z*) = lca*_T _*(*x*, *y*, *z*) we conclude that *S *must display ((σ (*x*), σ(*y*)), σ(*z*)) as *S *is assumed to be a species tree for (*T*,*t*, σ).

Now suppose that *t*(lca*_T _*(*x*, *y*)) = □ and therefore, *µ *(lca*_T _*(*x*, *y*)) ∈ *H*. Moreover, *µ *(lca*_T _*(*x*, *y*, *z*)) ∈ *W *holds. Hence, Lemma 3 and property *(iv) *together imply that $\text{lc}{\text{a}}_{S}\left(\sigma \left(x\right),\;\sigma \left(y\right)\right){≺}_{S}\mu \left(\text{lc}{\text{a}}_{T}\left(x,y\right)\right){≺}_{S}\mu \left(\text{lc}{\text{a}}_{T}\left(x,y,z\right)\right)$. Thus, we again obtain that the triple ((σ(*x*), σ(*y*)), σ(*z*)) is displayed by *S*. □

**Figure 3:**
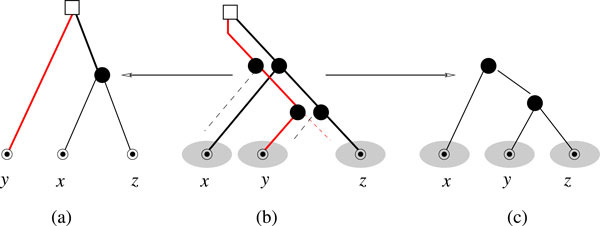
**Triples with duplication event at the root**. Triples from *T *whose root is a duplication event are in general not displayed from the species tree *S*. (a) Triple with duplication event at the root obtained from the true evolutionary history of *T *shown in panel (b). Panel (c) is the true species tree. In the triple (a) the species *y *appears as the outgroup even though the *x *is the outgroup in the true species tree.

It is important to note that a similar argument cannot be made for triples in ℜ(T) rooted in a duplication vertex of *T *as such triplets are in general not displayed by a species tree for (*T*,*t*, σ). We present the generic counterexample in Figure 3. To state our main result (Theorem 6), we require a further definition.

**Definition 5**. *For *(*T*,*t*, σ)*, we define the set*

(3) G=G(T,t,σ)={((a,b),c)|∃((x,y),z)∈G(T,t,σ)withσ(x)=a,σ(y)=b,andσ(z)=c}

As an immediate consequence of Lemma 4, G(T,t,σ) must be displayed by any species tree for (*T*,*t*, σ) with leaf set *B*.

**Theorem 6**. *Let S be a species tree with leaf set B. Then there exists a reconciliation map µ from *(*T*,*t*, σ) *to S whenever S displays all triples in *G(T,t,σ).

*Proof*. Recall that *L *is the leaf set of *T *= (*V*, *E*). Put *S *= (*W*, *H*) and G=G(T,t,σ). We first consider the subset G:={x∈V|t(x)∈{∙,⊙}} of *V *comprising of the leaves and speciation vertices of *T*.

We explicitly construct the map *µ *: *G *→ *W *as follows. For all *x *∈ *V *, we put

(Ml)μ(x)=σ(x)ift(x)=⊙,

(M2) *µ*(*x*) = lca*_S_*(σ(*L*(*x*))) if *t*(*x*) = •.

Note that alternative (M1) ensures that *µ *satisfies Condition *(i)*. Also note that in view of the simple consequence following the statement of Condition *(C) *we have for all *x *∈ *V *with *t*(*x*) = • that there are leaves *y*', *y*" ∈ *L*(*x*) with σ(*y*') ≠ σ(*y*"). Thus lca*_S_*(*µ*(*L*(*x*)) ∈ *W ***\ ***B*, i.e. *µ *satisfies Condition *(ii)*. Also note that, by definition, alternative (M2) ensures that *µ *satisfies Condition *(v)*.

**Claim: **If *x*, *y *∈ *G *with $x{≺}_{T}y$ then $\mu \left(x\right){≺}_{S}\mu \left(y\right)$.

Since *y *cannot be a leaf of *T *as $x{≺}_{T}y$ we have *t*(*y*) = •. There are two cases to consider, either *t*(*x*) = • or t(x)=⊙. In the latter case *µ*(*x*) = σ(*x*) ∈ *B *while *µ*(*y*) ∈ *W ***\ ***B *as argued above. Since *x *∈ *L*(*y*) we have $\mu \left(x\right){≺}_{S}\mu \left(y\right)$, as desired.

Now suppose *t*(*x*) = •. Again by the simple consequence following Condition *(C)*, there are leaves *x*', *x*" ∈ *L*(*x*) with *a *= *σ*(*x*') *≠ σ*(*x*") = *b*. Since $x{≺}_{T}y$ and *t*(*y*) = •, by Condition *(C)*, we conclude that *c *= *σ*(*y*') ∉ *σ*(*L*(*x*)) holds for all *y*' ∈ *L*(*y*) **\ ***L*(*x*). Thus,((a,b),c)∈G. But then ((*a*, *b*), *c*) is displayed by *S *and therefore $\text{lc}{\text{a}}_{S}\left(a,\;b\right){≺}_{S}\text{lc}{\text{a}}_{S}\left(a,\;b,\;c\right).$. Since this holds for all triples $\left(\left(x^{!},x^{\prime \prime }\right),y^{\prime }\right)\in G$ with *x*', *x*" ∈ *L*(*x*) and *y*' ∈ *L*(*y*) **\ ***L*(*x*) we conclude $\mu \left(x\right)=1\text{c}{\text{a}}_{S}\left(\sigma \left(L\left(x\right)\right)\right){≺}_{S}\text{lc}{\text{a}}_{S}\left(\sigma \left(L\left(x\right)\right)\cup \sigma \left(L\left(y\right)\backslash L\left(x\right)\right)\right)=1\text{c}{\text{a}}_{S}\left(\sigma \left(L\left(y\right)\right)\right)=\mu \left(y\right),$ establishing the claim. It follows immediately that *µ *also satisfies Condition *(iv.2) *if *x *and *y *are contained in *G*.

Next, we extend the map *µ *to the entire vertex set *V *of *T *using the following observation. Let *x *∈ *V *with *t*(*x*) = □. We know by Lemma 3 that *µ*(*x*) is an edge [*u*, *v*] ∈ *H *so that $\text{lc}{\text{a}}_{S}\left(\sigma \left(L\left(x\right)\right)\right){≼}_{S}v$. Such an edge exists for *v *= lca*_S_*(σ(*L*(*x*))) by construction. Every speciation vertex *y *∈ *V *with $x{≺}_{T}y$ therefore necessarily maps above this edge, i.e., $u{≼}_{S}\mu \left(y\right)$ must hold. Thus we set

(M3) *µ*(*x*) = [*u*, lca*_S_*(σ(*L*(*x*)))] if *t*(*x*) = □.

which now makes *μ *a map from *V *to *W *∪ *H*.

By construction, Conditions *(iii)*, *(iv.2) *and *(v) *are thus satisfied by *μ*. On the other hand, if there is a speciation vertex *y *between two duplication vertices *x *and *x*' of *T *, i.e., $x{≺}_{T}y{≺}_{T}x^{\prime }$, then $\mu \left(x\right){≺}_{S}\mu \left(x^{\prime }\right)$. Thus *μ *also satisfies Condition *(iv.1)*.

It follows that *μ *is a reconciliation map from (*T*,*t*, σ) to *S*. □

**Corollary 7**. *Suppose that S is a species tree for *(*T*,*t*, *σ*) *and that L and B are the leaf sets of T and S, respectively*. *Then a reconciliation map μ from *(*T*,*t*, *σ*) *to S can be constructed in O*(|*L***||***B|*).

*Proof*. In order to find the image of an interior vertex *x *of *T *under *μ*, it suffices to determine *σ *(*L*(*x*)) (which can be done for all *x *simultaneously, e.g. by bottom up transversal of *T *in *O*(|*L***||***B|*) time) and lca*_S_*(σ(*L*(*x*))). The latter task can be solved in linear time using the idea presented in [39] to calculate the lowest common ancestor for a group of nodes in the species tree. □

We remark that given a species tree *S *on *B *that displays all triples in G(T,t,σ), there is no freedom in the construction of a reconciliation map on the set {x∈V|t(x)∈{∙,⊙}}. The duplication vertices of *T*, however, can be placed differently, resulting in possibly exponentially many reconciliation maps from (*T*,*t*, σ) to *S*.

Lemma 4 implies that consistency of the triple set G(T,t,σ) is necessary for the existence of a reconciliation map from (*T*,*t*, σ) to a species tree on *B*. Theorem 6, on the other hand, establishes that this is also sufficient. Thus, we have

**Theorem 8**. *There is a species tree on B for *(*T*,*t*, **σ**) *if and only if the triple set *G(T,t,σ)*is consistent*.

We remark that a related result is proven in [26, Theorem.5] for the full tree reconciliation problem starting from a forest of gene trees.

It may be surprising that there are no strong restrictions on the set G(T,t,σ) of triples that are implied by the fact that they are derived from a gene tree (*T*,*t*, σ).

**Theorem 9**. *For every set *–x*of triples on some finite set B of size at least one there is a gen e tree T *= (*V*, *E*) *with leaf set L together with an event map *t:V→{∙,□,⊙}*and a map σ *: *L ***→ ***B that assigns to every leaf of T the species in B it resides in, such that *–x=G(T,t,σ).

*Proof*. Irrespective of whether –x is consistent or not we construct the components of the required 3-tuple (*T*,*t*, *σ*) as follows: To each triple $r_{k}=\left(\left(x_{k1},x_{k2}\right),x_{k3}\right)\in –x$ we associate a triple $T_{k}=\left(\left(a_{k1},a_{k2}\right),a_{k3}\right)$ via a map $\sigma_{k}:L_{k}=\left\{a_{k1},a_{k2},a_{k3}\right\}\to \left\{x_{k1},x_{k2},x_{k3}\right\}$ with $\sigma \left(a_{ki}\right)=x_{ki}$ for *i *= 1, 2, 3 where we assume that for any two distinct triples $r_{k},r_{l}\in –x$ we have that σ*_k_*(*L_k_*) ∩ σ*_l_*(*L_l _*) = 0̸. Then we obtain *T *= (*V*, *E*) by first adding a single new vertex *ρ_T _*to the union of the vertex sets of the triples *T_k _*and then connecting *ρ_T _*to the root *ρ_k _*of each of the triples *T_k_*. Clearly, *T *is a phylogenetic tree on $L=\cup_{r_{k}\in –x}L\left(\rho_{k}\right)$. Next, we define the map t:V→{∙,□,⊙} by putting *t*(*ρ_T _*) = □, t(a)=⊙ for all *a *∈ *L *and *t*(*a*) = • for all *a *∈ *V ***− **(*L *∪ **{***ρ _T _***}**). Finally, we define the map *σ *: *L ***→ ***B *by putting, for all *a *∈ *L*, σ(*a*) = σ*_k _*(*a*) where *a *∈ *L_k_*. Clearly G(T,t,σ)=–x.    □

**Figure 4:**
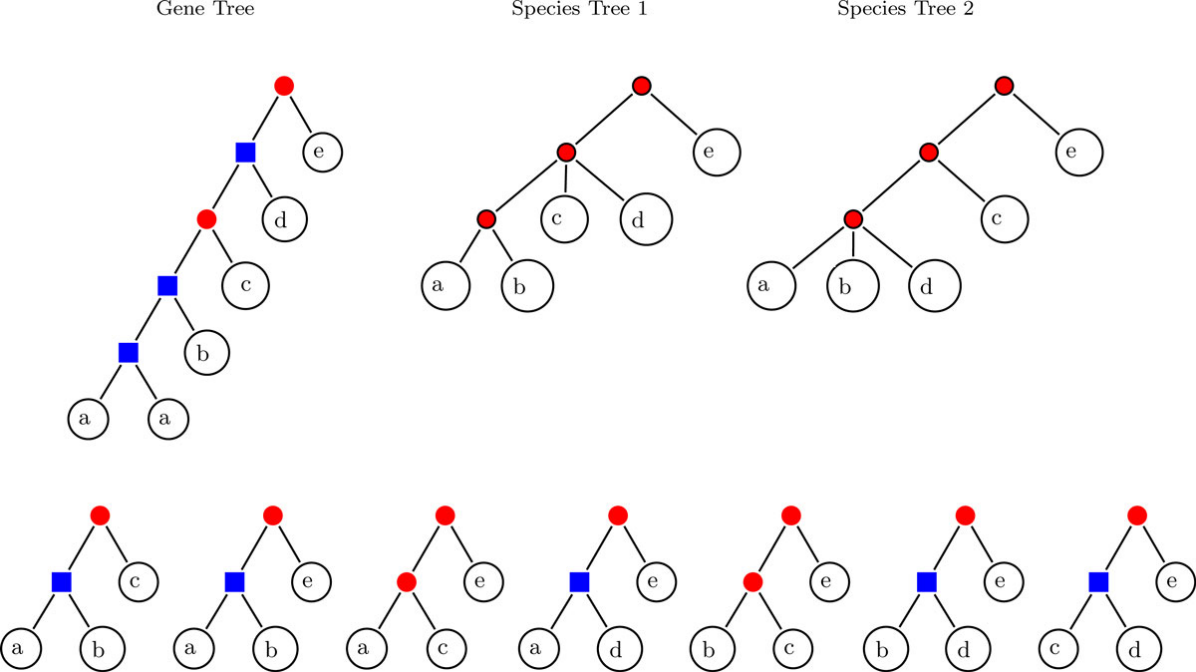
**Inferred species trees**. The set G(T,t,σ) inferred from the event labeled gene tree (*T*,*t*, σ) does not necessarily define a unique species tree. For clarity of exposition, we have identified, via the map σ, the leaves of the gene tree and of the set of triples G(T,t,σ) with the species they reside in.

We remark that the gene tree constructed in the proof of Theorem 9 can be made into a binary tree by splitting the root *ρ_T _*into a series of duplication and loss events so that each subtree is the descendant of a different paralog. Since by Theorem. 9 there are no restrictions on the possible triple sets G(T,t,σ), it is clear that *S *will in general not be unique. An example is shown in Figure 4.

#### Results for simulated gene trees

In order to determine empirically how much information on the species tree we can hope to find in event labeled gene trees, we simulated species trees together with corresponding event-labeled gene trees with different duplication and loss rates. Approximately 150 species trees with 10 to 100 species were generated according to the "age model" [40]. These trees are balanced and the edge lengths are normalized so that the total length of the path from the root to each leaf is 1. For each species tree, we then simulated a gene tree as described in [41], with duplication and loss rate parameters *r *∈ 0[1] sampled uniformly. Events are modeled by a Poisson distribution with parameter *r *· *ℓ*, where *ℓ *is the length of an edge as generated by the age model. Losses were additionally constrained to retain at least one copy in each species, i.e., σ(*L*) = *B *is enforced. After determining the triple set G(T,t,σ) according to Theorem 6, we used BUILD [27] (see also [42]) to compute the species tree. In all cases BUILD returns a tree that is a homomorphic contraction of the simulated species tree. The difference between the original and the reconstructed species tree is thus conveniently quantified as the difference in the number of interior vertices. Note that in our situation this is the same as the split metric [27].

**Figure 5:**
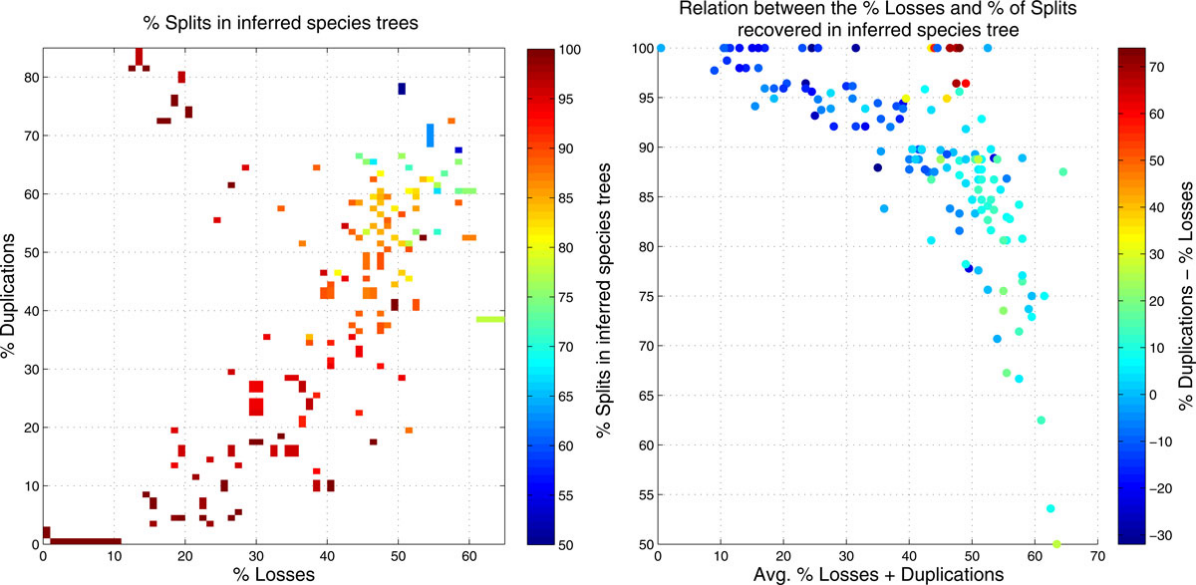
**Recovered splits in species trees**. **Left: **Heat map that represents the percentage of recovered splits in the inferred species tree from triples obtained from simulated event-labeled gene trees with different loss and duplication rates. **Right: **Scattergram that shows the average of losses and duplications in the generated data and the accuracy of the inferred species tree.

The results are summarized in Figure 5. Not surprisingly, the recoverable information decreases in particular with the rate of gene loss. Nevertheless, at least 50% of the splits in the species tree are recoverable even at very high loss rates. For moderate loss rates, in particular when gene losses are less frequent than gene duplications, nearly the complete information on the species tree is preserved. It is interesting to note that BUILD does not incorporate splits that are not present in the input tree, although this is not mathematically guaranteed.

## Discussion

Event-labeled gene trees can be obtained by combining the reconstruction of gene phylogenies with methods for orthology detection. Orthology alone already encapsulates partial information on the gene tree. More precisely, the orthology relation is equivalent to a homomorphic image of the gene tree in which adjacent vertices denote different types of events. We discussed here the properties of reconciliation maps *μ *from a gene tree *T *along with an event labelling map *t *and a gene to species assignment map σ to a species tree *S*. We show that (*T*,*t*) event labeled gene trees for which a species tree exists can be characterized in terms of the set σ of triples that is easily constructed from a subset of triples of *T*. Simulated data shows, furthermore, that such trees convey a large amount of information on the underlying species tree, even if the gene loss rate is high.

It can be expected that for real-life data the tree *T *contains errors so that G:=G(T,t,σ) may not be consistent. In this case, an approximation to the species tree could be obtained e.g. from a maximum consistent subset of G . Although (the decision version of) this problem is NP-complete [43,44], there is a wide variety of practically applicable algorithms for this task, see [35,45]. Even if G  is consistent, the species tree is usually not uniquely determined. Algorithms to list all trees consistent with G  can be found e.g. in [46,47]. A characterization of triple sets that determine a unique tree can be found in [48]. Since our main interest is to determine the constraints imposed by (*T*,*t*, σ) on the species tree *S*, we are interested in a least resolved tree *S *that displays all triples in G . The BUILD algorithm and its relatives in general produce minor-minimal trees, but these are not guaranteed to have the minimal number of interior nodes. Finding a species tree with a minimal number of interior nodes is again a hard problem [37]. At least, the vertex minimal trees are among the possibly exponentially many minor minimal trees enumerated by Semple's algorithms [36].

For a given species tree *S*, it is rather easy to find a reconciliation map *μ *from (*T*,*t*, σ) to *S*. A simple solution *μ *is closely related to the so-called LCA reconcilation: every node *x *of *T *is mapped to the last common ancestor of the species below it, lca*_S_*(σ(*L*(*x*))) or to the edge immediately above it, depending on whether *x *is speciation or a duplication node. While this solution is unique for the speciation nodes, alternative mappings are possible for the duplication nodes. The set of possible reconciliation maps can still be very large despite the specified event labels. If the event labeling *t *is unknown, there is a reconciliation from any gene tree *T *to any species tree *S*, realized in particular by the LCA reconciliation, see e.g. [26,38]. The reconciliation then defines the event types. Typically, a parsimony rule is then employed to choose a reconciliation map in which the number of duplications and losses is minimized, see e.g. [1,4,5,9]. In our setting, on the other hand, the event types are prescribed. This restricts the possible reconciliation maps so that the gene tree cannot be reconciled with an arbitrary species tree any more. Since the observable events on the gene tree are fixed, the possible reconciliations cannot differ in the number of duplications. Still, one may be interested in reconciliation maps that minimize the number of loss events. An alternative is to maximize the number of duplication events that map to the same edge in *S *to account for whole genome and chromosomal duplication events [9].

## Conclusions

Our approach to the reconciliation problem via event-labeled gene trees opens up some interesting new avenues to understanding orthology. In particular, the results in this contribution combined with those in [22] concerning cographs should ultimately lead to a method for automatically generating orthology relations that takes into account species relationships without having to explicitly compute gene trees. This is potentially very useful since gene tree estimation is one of the weak points of most current approaches to orthology analysis.

## Competing interests

The authors declare that they have no competing interests.

## Authors' contributions

All authors contributed to the development of the theory. MHR and NW produced the simulated data. All authors contributed to writing, reading, and approving the final manuscript.
